# Genetically encoded optical activation of DNA recombination in human cells†
†Electronic supplementary information (ESI) available: Experimental protocols. See DOI: 10.1039/c6cc03934k



**DOI:** 10.1039/c6cc03934k

**Published:** 2016-06-09

**Authors:** J. Luo, E. Arbely, J. Zhang, C. Chou, R. Uprety, J. W. Chin, A. Deiters

**Affiliations:** a Department of Chemistry , University of Pittsburgh , 219 Parkman Ave , Pittsburgh , Pennsylvania 15260 , USA . Email: deiters@pitt.edu; b Medical Research Council Laboratory of Molecular Biology , Francis Crick Ave , Cambridge CB20QH , UK; c Department of Chemistry and The National Institute for Biotechnology in the Negev , Ben-Gurion University of the Negev , Beer-Sheva , 84105 , Israel; d Department of Chemistry , North Carolina State University , Raleigh , North Carolina 27695 , USA

## Abstract

We developed two tightly regulated, light-activated Cre recombinase enzymes through site-specific incorporation of two genetically-encoded photocaged amino acids in human cells. Excellent optical off to on switching of DNA recombination was achieved. Furthermore, we demonstrated precise spatial control of Cre recombinase through patterned illumination.

## Abstract 2

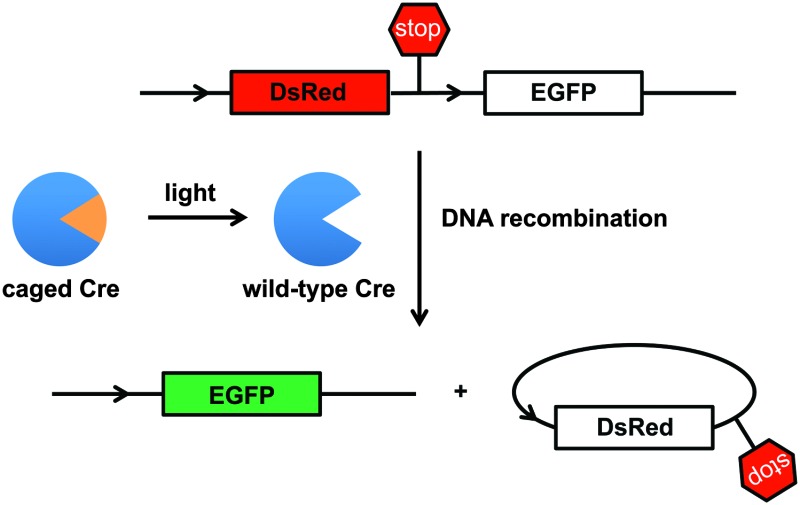
Two precisely regulated, light-activated Cre recombinase enzymes were generated through the site-specific incorporation of two genetically encoded photocaged amino acids in human cells.

DNA recombination is an important biological process that is commonly used for the activation and deactivation of gene expression in single cells and multicellular organisms, and thus has been engineered into a versatile genome manipulation tool.^1^ Cre recombinase, a 38 kD protein from bacteriophage P1^2^ that recognizes *lox*P sites on dsDNA, is the most widely applied recombinase. *loxP* sites consist of two 13-base pair repeats flanking an 8-base pair asymmetric sequence. Cre recombinase targets the *loxP* sites, cleaves the dsDNA and either inserts or excises sequences through a double recombination event that proceeds *via* a Holiday junction.^3^ The Cre-*loxP* recombination system has proven to be a robust and reliable site-specific recombination tool due to its efficient function in several organisms, including *E. coli*,^4^ yeast,^5^ mice,^6–8^ zebrafish,^9^ and drosophila.^10^ Moreover, the Cre-*loxP* system is active on any type of DNA, such as linear, supercoiled, or circular,^11^ and has been extensively applied in genome engineering, enabling efficient conditional gene knock-out and knock-in for functional genetics studies.^1^

Furthermore, conditional spatio-temporal control over the Cre recombination system enables activation and deactivation of gene function with enhanced precision. Initial attempts to temporally regulate Cre expression used inducible promoters^6,12–14^ and fusion proteins with ligand binding domains, such as the rapamycin inducible FKBP-FRB dimerization pair or the estrogen receptor (ER).^15,16^ However, triggering Cre function with small molecules limits the ability to achieve spatial control. In order to address this limitation, three different light-activation strategies were developed: (1) the two fragments of a split Cre recombinase were fused to cryptochrome 2 (CRY2) and *Arabidopsis* CIB1 (cryptochrome-interacting basic-helix–loop–helix), and exposure to blue light (450 nm) induced dimerization of CIB1 and CRY2 and Cre activation.^17–19^ However, the recombination activity was limited, requiring extended light exposure. (2) The small molecule inducible systems were expanded by introducing photocaged tamoxifen and rapamycin analogs for photochemical control of DNA recombination.^12,20–23^ However, limited recombination activity was observed after light exposure and the diffusible small molecule ligand could induce off-target effects. (3) A caged Cre enzyme was expressed in *E. coli*, isolated and purified, and transfected into mammalian cells.^24^ This enabled spatio-temporal activation of DNA recombination in tissue culture, but the required enzyme isolation, purification, and protein transfection limit its application.

Here, we address limitations of the aforementioned approaches by reporting a tightly regulated, light-activated Cre recombinase that is genetically encoded in mammalian cells. We are utilizing the *Methanosarcina barkeri* or *Methanosarcina mazei* pyrrolysyl tRNA synthetase/tRNA_CUA_ (PylRS/tRNA_CUA_) pairs,^25^ which we have previously been used to optically control transcription, nuclear localization, CRISPR/Cas9, and kinase function.^26–30^ This system allows for the site-specific incorporation of photocaged amino acids into proteins in mammalian cells, enabling the genetic encoding of light-activated processes.

Cre has a catalytic tyrosine (Y324) that is crucial for formation of a covalent protein–DNA intermediate, and thus catalyzes sequential strand exchange among the cognate *loxP* sites, and the ability to photocage this amino acid residue was previously demonstrated through unnatural amino acid mutagenesis in *E. coli*.^24^ In order to generate a light-activated Cre recombinase in an eukaryotic system, we first selected the catalytic Tyr324 as a target (Fig. 1B), by genetically encoding the incorporation of the caged tyrosine **ONBY** (Fig. 1A) using a specifically engineered PylRS mutant, ONBYRS.^31^ The *ortho*-nitrobenzyl-based photolabile protecting groups are the most commonly used protecting groups for the photocontrol of biological processes in living cells. Live cell applications require specific photochemical properties from the protecting group: (i) complete stability under physiological conditions; (ii) efficient photolysis to ensure complete photodeprotection using minimal light exposure; (iii) photodeprotection at wavelengths >350 nm to minimize phototoxicity; (iv) photolysis byproducts that are non-reactive and non-toxic.

**Fig. 1:**
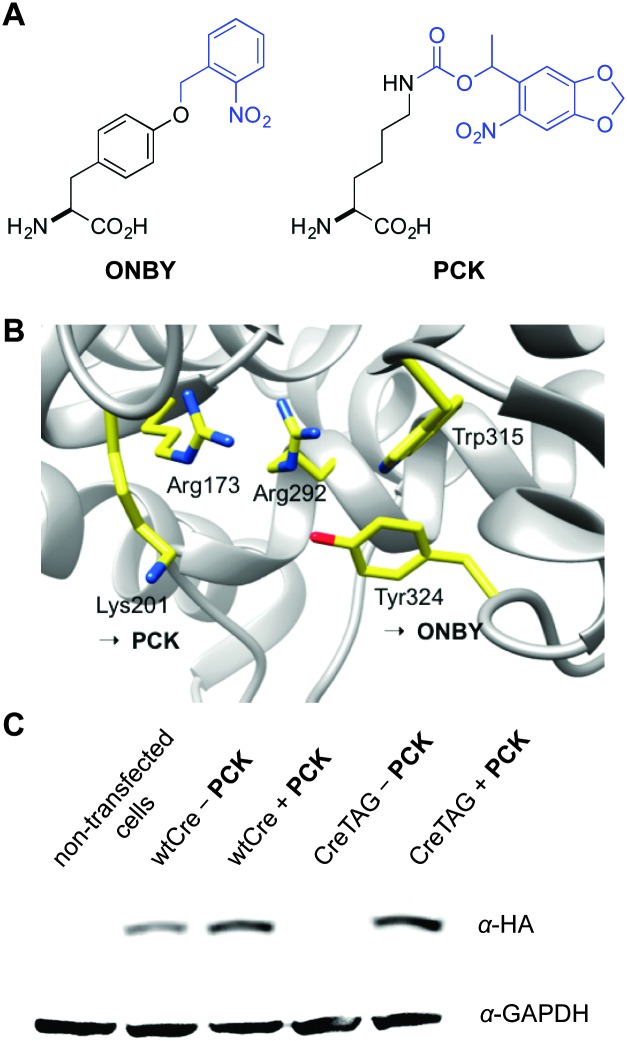
(A) Structure of photocaged tyrosine (**ONBY**) and photocaged lysine (**PCK**). (B) Active site residues of Cre recombinase, including the essential K201 and Y324 (PDB ; 1Q3V). (C) Anti-HA Western blot showing amino acid dependent expression of caged Cre recombinase in HEK293T cells in the absence or presence of **PCK** (1.0 mM). An anti-GAPDH Western blot is shown as a loading control. Expression levels of CreK201**PCK** are similar to wild-type Cre recombinase expression.

A nuclear targeted and affinity-tagged Cre recombinase was generated through amplification of the Cre gene from pET21-Cre^24^ and cloning into the pCS2HA-NLS backbone, creating pCS2HA-NLS-Cre.^32^ A TAG codon was introduced at position 324 and the mutated Cre gene (HA-NLS-CreY324TAG) was cloned into the ONBYRS expression vector,^31^ creating the pONBYRS-HA-NLS-CreY324TAG plasmid. As a convenient reporter of cellular Cre activity, the Cre Stoplight plasmid (Cre-Stoplight) was used (Fig. 2A).^24^ Cre-Stoplight encodes DsRed and a transcription termination region, both flanked by *loxP* sites and located upstream of an EGFP gene.^33^ In the absence of Cre-mediated recombination, only DsRed is expressed; after recombination, the *loxP* flanked DsRed coding region is excised and the EGFP coding region is placed under control of the CMV promoter. Thus, in the presence of active Cre recombinase, the expression of DsRed is turned off while the expression of EGFP is turned on, leading to green fluorescent cells.

**Fig. 2:**
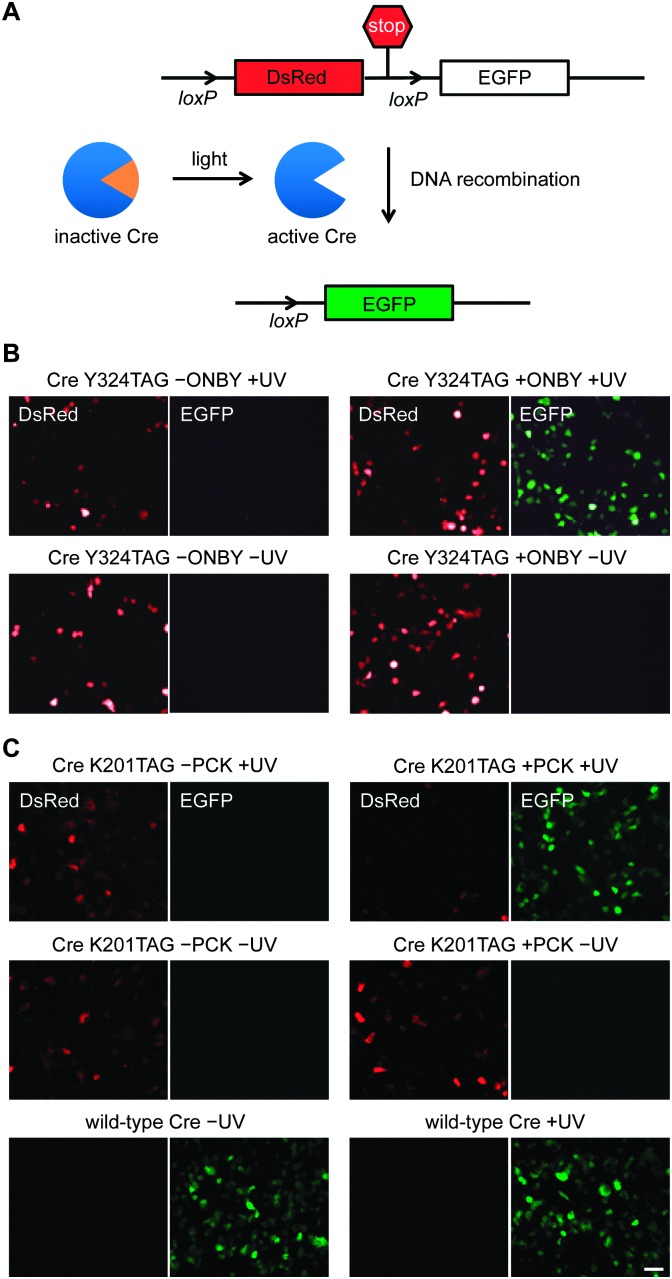
(A) The Cre-Stoplight reporter encodes for DsRed followed by a transcription termination signal that is flanked by *loxP* sites. Light-activation of caged Cre recombinase results in Cre-mediated recombination, and activation of EGFP expression through excision of the DsRed-terminator cassette. (B and C) Fluorescence microscopy images of HEK293T cells expressing the Cre-Stoplight reporter and the caged Cre recombinases Y324**ONBY** or K201**PCK**. The caged enzymes are completely inactive until UV exposure (365 nm) triggers protein decaging, enzymatic activity, and DNA recombination. Scale bar represents 50 μm.

We then demonstrated genetically encoded photocontrol of Cre bearing **ONBY** at position 324 in HEK293T cells. Cells were co-transfected with the Cre-Stoplight reporter plasmid, pONBYRS-CreY324TAG, and p4CMVE-U6-PylT (encoding four copies of the pyrrolysine tRNA_CUA_),^27^ and grown in the absence or presence of **ONBY** (0.4 mM). Illumination of cells grown in the presence of ONBY for 5 minutes led to a substantial increase in expression of EGFP, as determined by fluorescence microscopy (Fig. 2B). This is consistent with the photoactivation of Cre recombinase and the activation of EGFP transcription by the excision of the transcriptional terminator that precedes it in a Cre-*loxP* mediated process. Control experiments in which **ONBY**, light, or both **ONBY** and light were omitted did not lead to any activation of EGFP expression, demonstrating tight and background-free optical control of Cre recombinase activity.

While the simple *ortho*-nitrobenzyl group in **ONBY** can be successfully applied, other photo-labile protecting groups, such as the methylated 6-nitropiperonylmethyl group in photocaged lysine (**PCK**, Fig. 1A), are even more suitable for live cell applications. Electron-donating substituents on the aromatic chromophore in **PCK** result in a bathochromic shift of the absorption maximum (and the effective decaging wavelength) and consequently lower phototoxicity. In addition, methyl substitution at the benzylic position improves decaging kinetics and the biocompatibility of the protecting group since it transforms the photolysis product from an aldehyde to a less reactive ketone. In addition, **PCK** shows improved solubility (>10 mM) compared to **ONBY** (≤0.5 mM) in cell culture media.

Thus, we decided to test the genetic encoding of an improved light-activated Cre recombinase through incorporation of **PCK** at position K201 within the active site of the enzyme (Fig. 1B). We selected this lysine as a target for caging group introduction, since it is highly conserved in almost all of the 80 Int family recombinases^34^ and since it is essential for catalytic activity as shown by the enzymatic inactivity of the K201A mutant.^35,36^ The side-chain of K201 is directed into the minor groove near the cleavage site of the DNA, providing interactions with the N3 of the +1 guanine base, 5′-O and 4′-O of the –1 sugar, and a tightly bound water molecule within hydrogen bonding distance.^36^ The NLS-HA-Cre gene, described above, was inserted into the CMV promoter-driven coding region of the pCKRS vector, which contains a PylRS evolved for **PCK**, generating pCKRS-NLS-HA-Cre. A TAG codon was then introduced at position K201, creating pCKRS-HA-NLS-CreK201TAG. The pCKRS-NLS-HA-Cre or pCKRS-NLS-HA-CreK201TAG plasmid was co-transfected with the p4CMVE-U6-PylT plasmid into HEK 293T cells in the absence or presence of **PCK** (1 mM) and a Western blot was performed (Fig. 1C). For wild-type expression, a band corresponding to a 39 kDa HA-tagged Cre recombinase was detected. In the case of caged Cre recombinase expression, the corresponding band was only observed in the presence but not the absence of **PCK**. This demonstrates that **PCK** was successfully incorporated into the mutant Cre protein with high fidelity. In addition, this indicates the improved expression efficiency of CreK201**PCK** compared to CreY324**ONBY**, as we were unable to obtain suitable Western blot results for the latter protein.

To demonstrate photoactivation of K201-caged Cre recombinase in live cells, HEK293T cells were co-transfected with the above described expression system and the Cre-Stoplight reporter plasmid.^31^ As expected, the expression of wild-type Cre recombinase led to intracellular recombination and observation of EGFP expression (Fig. 2C). As a negative control, the expression of caged Cre in the absence of **PCK** exclusively showed DsRed expression, indicating that no functional Cre recombinase was generated. This is further supported by Western blot analysis (Fig. 1C). Importantly, cells transfected with pCKRS-NLS-HA-CreTAG in the presence of **PCK** but in the absence of UV irradiation exclusively showed DsRed expression, verifying the complete catalytic inactivity of the caged Cre recombinase containing the K201 → **PCK** mutation. The protein was activated through a brief UV exposure (4 min, 365 nm), which induced decaging and subsequent Cre-catalyzed DNA recombination, activating EGFP expression.

In order to quantify the observed recombination activity of caged Cre recombinase in the absence or presence of UV exposure, imaging cytometry was conducted and the number of DsRed- and EGFP-positive cells were counted in six randomly selected areas for each well (Fig. 3A). The results showed no DNA recombination activity of the caged enzyme in the absence of UV exposure and a light-triggered OFF → ON switching that delivered enzymatic activity at almost the wild-type Cre recombinase level.

**Fig. 3:**
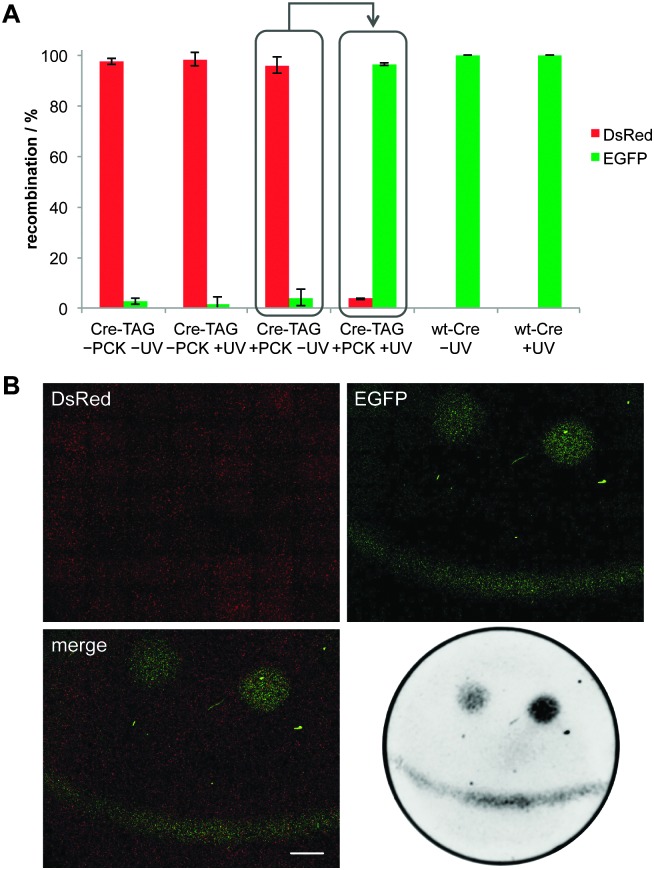
(A) Imaging cytometry of fluorescent cells from the light-activation of CreK201**PCK** (see Fig. 2C). Cells were counted in both the DsRed and EGFP channel over six randomly selected fields of view per well and counts were normalized to the total number of fluorescent cells (Nikon Elements). Error bars represent standard deviations from three independent experiments. (B) Spatial control of DNA recombination through patterning of a smiley face onto a layer of HEK293T cells in a 35 mm imaging μ-dish (observation area 21 mm, ibidi) expressing caged Cre recombinase and the Cre-Stoplight reporter. The left eye and mouth area were exposed to 365 nm light for 3.5 min, while the right eye was exposed for 5 min, which led to increased recombination activity. DsRed and EGFP channels were imaged through tiling (7 × 7, 4× objective, Nikon A1R microscope). Scale bar represents 2 mm. For improved contrast, fluorescence was also imaged using a Bio-Rad ChemiDoc system.

To demonstrate spatial control over DNA recombination in mammalian cells, HEK293T cells expressing both the caged Cre recombinase and the Cre-Stoplight reporter were exposed to patterned irradiation. After light exposure, the cells were incubated for 24 h and fluorescence in live cells was imaged (Fig. 3B). DsRed fluorescence was detected in all transfected cells but EGFP fluorescence was only observed in the areas exposed to localized irradiation showing tight spatial control over the DNA recombination event. In addition, fine-tuning of the DNA recombination in response to different exposure times was demonstrated by irradiating certain areas for different durations (in the same experiment and the same plate). Longer UV treatment (5 min) led to greater Cre activation and increased EGFP expression (see left *versus* right eye in Fig. 3B).

In summary, we have engineered a genetically encoded, light-activated Cre recombinase in mammalian cells. The activity of the enzyme can be stringently regulated both spatially and temporally through the use of a light-removable caging group installed directly on the essential residues K201 or Y324 in the active site. The overall efficiency of light-activation of DNA recombination was significantly improved over other photoresponsive Cre systems and enabled the spatial control of gene function.^12,17,20–22,24^ By applying an engineered pyrrolysyl tRNA synthetase/tRNA system for the genetic encoding of a photocaged lysine or tyrosine, the developed Cre recombinase system can be easily adapted to other eukaryotic cells and multicellular model organisms.^37^ The use of lysine protected with a substituted *ortho*-nitrobenzyl protecting group (compared to the unsubstituted **ONBY**) provides improved biocompatibility and improved expression levels of the caged Cre protein, which enables future use of the described system in numerous applications, including optical control in tissue xenografts, the creation of knock-in and knock-out organisms,^38^ and the potential to designate stem cell fate and pluripotency induction in a spatio-temporal fashion.^39^ The targeted, highly conserved lysine site can also be found in other Int family proteins, such as FLP recombinase, XerD resolvase, λ phage integrase, and transposase, further expanding this approach in future applications in mammalian cells.^36^

This research was supported by the Charles E. Kaufman Foundation of the Pittsburgh Foundation. We thank the Hughes lab (MSU) for the Cre Stoplight plasmid.
