# Supplementary material for: Genetically encoded optical activation of DNA recombination in human cells
Source: Chem Commun (Camb). 2016 Jun 9;52(55):8529–32. doi: 10.1039/c6cc03934k (PMC5048445; doi:10.1039/c6cc03934k)
Supplement: Supplementary file 1 [file cc-052-C6CC03934K-s001.pdf]

Electronic Supplementary Material (ESI) for ChemComm.
This journal is © The Royal Society of Chemistry 2016

Supporting information

Genetically Encoded Optical Activation of DNA Recombination in Human Cells

Ji Luo,[1] Eyal Arbely,[2,3] Jie Zhang,[4] Chungjung Chou,[4] Rajendra Uprety,[4] Jason W. Chin,[2] and Alexander Deiters*,[1]

[1] *Department of Chemistry, University of Pittsburgh, Pittsburgh, Pennsylvania 15260, United States*

[2] *Medical Research Council Laboratory of Molecular Biology, Francis Crick Avenue, Cambridge CB20QH, United Kingdom*

[3] *Department of Chemistry and The National Institute for Biotechnology in the Negev, Ben-Gurion University of the Negev, Beer-Sheva, 84105, Israel*

[4] *Department of Chemistry, North Carolina State University, Raleigh, North Carolina 27695, United States*

pCKRS-HA-Cre vector construction: GC5 competent cells (Invitrogen) were used for cloning and sub-cloning experiments. The Cre gene was PCR amplified from pET21-Cre[1] using the primers 5'-AGAGAGGCTAGCTCCAATTTACTGACCGTACACCAA-3' and 5'-AGAGAGCTCGAGATCGCCATCTTCCAGCAGGCGCACCATTGCCCCTGT-3', which contain KpnI and XbaI restriction sites, respectively. The Cre gene was then inserted into pCS2 as a KpnI and XbaI fragment, generating pCS2_NLS-HA-Cre, and yielding HA-tagged wild-type Cre recombinase upon expression in mammalian cell. The expected vector sequence was confirmed by DNA sequencing using the internal sequencing primers 5'-CTGATTTCGACCAGGTTCGT-3' and 5'-GCTAACCAGCGTTTTCGTTC-3'. The HA-Cre gene was the amplified using the primers 5'-AGAGAGGCTAGCTCCAATTTACTGACCGTACACCAA-3' and 5'-AGAGAGCTCGAGATCGCCATCTTCCAGCAGGCGCACCATTGCCCCTGT-3', which contain NheI and MfeI restriction sites for ligation into pCKRS. The expected vector sequence was confirmed by DNA sequencing using the internal sequencing primers 5'-CTGATTTCGACCAGGTTCGT-3' and 5'-GCTAACCAGCGTTTTCGTTC-3'.

pCKRS-HA-CreKTAG vector mutagenesis for PCK incorporation. Following the manufacturer's protocol (Invitrogen), a QuikChange lightening kit was used to mutate K201 to an amber stop codon (TAT→TAG) with primers 5'-GGACCAATGTAAATATTGTCATGAACTAGATCC-3' and 5'-ATCCAGGTTACGGATCTAGTTCATGACAATATT-3', creating pCKRS-HA-CreTAG. Successful amber stop codon mutation was confirmed by DNA sequencing using the internal Cre sequencing primer 5'-CTGATTTCGACCAGGTTCGT-3'.

pONBYRS-CreY324TAG vector construction: The gene coding for CreY324TAG was amplified from plasmid pET21-CreY324TAG[1] using primers 5'-AGAGAGGCTAGCATGTCC AATTTACTGACCGTACAC-3' and 5'-TAACAACAACAATTGTTATCAGTGGTGGTGGTGGT GGTGCTC-3', digested with NheI and MfeI and subcloned in plasmid pMmONBYRS-mCherry-TAG-EGFP-HA.[2]

Western blots. HEK 293T cells were passaged into 6-well plates, and grown to 80% confluence. The cells were then transfected with the pCKRS_NLS_HA_Cre or pCKRS_NLS_HA_CreKTAG and p4CMVE-U6-PylT (2 μg each plasmid per well) using linear Polyethylenimine (PEI) transfection reagent in antibiotic free DMEM medium (Invitrogen) with 10% FBS. The transfection was incubated at 37 °C overnight, The cells were gently washed with ice-cold PBS and lysed with NE-PER nuclear protein extraction kit (Thermo Scientific) following the manufacturer's protocol. The protein extraction was analyzed on a 10% SDS-PAGE and transferred to a PVDF membrane (80 V, 1.5 h). The membrane was incubated in blocking buffer (5% nonfat milk/TBST (0.2% Tween 20, 20 mM Tris–HCl, 150 mM NaCl, pH 7.4) for 30 min and washed three times with TBST buffer. The membrane was then incubated with the primary antibody, α-HA-probe (Y-11) rabbit polyclonal IgG (sc-805, Santa Cruz Biotech), overnight at 4 °C, rinsed and incubated with a fluorescent secondary antibody, goat-α-rabbit IgG-Cy3 antibody (GE Healthcare), for 1 h at room temperature. Cy3 imaging was performed with a Typhoon FLA7000 scanner (GE Healthcare). The other blots as a loading control were probed and incubated with the primary GAPDH antibody (G-9) mouse monoclonal IgG_1 (sc-365062, Santa Cruz Biotech) overnight at 4 °C, followed by a goat anti-mouse IgG-HRP secondary antibody (sc-2031, Santa Cruz Biotech) for 1 h at room temperature. The blots were washed by TBST for three times, and then incubated in working solution (Thermo Scientific SuperSignal West Pico Chemiluminescent substrate) for 5 min. The blots were imaged by ChemiDoc (BioRad).

Photoactivation of CreY324ONBY. HEK-293 cells were grown in Dubelcco's modified Eagle's medium (DMEM) + GlutaMAX (Gibco) containing 10% fetal bovine serum and antibiotics, at 37°C in 5% CO_2 atmosphere. Before transfection, medium was replaced with antibiotics free medium supplemented, where indicated, with 0.4 mM of **ONBY**. Transfections were carried out with Lipofectamine 2000 using 0.2 μg of Cre-Stoplight plasmid, 0.4 μg of p4CMVE-U6-PylT and 0.6 μg of pONBYRS-CreY324TAG. After 24 h's post transfection, medium was replaced with fresh DMEM without **ONBY**, cells incubated for 2 h at 37°C in 5% CO_2 atmosphere and where indicated, exposed to 365 nm light for 5 min using 365 nm LED array, positioned below the plate. Expression level of EGFP and DsRed was measured 24 h after photo-deprotection.

Photoactivation of CreK201PCK. HEK 293T cells were plated in a 96-well cell culture (Greiner) microplate and grown to 75% confluency in Dubelcco's modified Eagle's medium (DMEM) supplemented with 10% FBS and 1% penicillin/streptomycin at 37 °C in 5% CO_2 atmosphere. The cells were co-transfected with the pCKRS-Cre or pCKRS-CreK201TAG, p4CMVE-U6-PylT, and the pC-SL reporter plasmid (100 ng of each) using linear PEI (2 μL per well) in the presence or absence of **PCK** (1 mM) overnight at 37 °C. The media was replaced with fresh DMEM without **PCK**, and cells were incubated for 2 h at 37 °C in 5% CO_2 atmosphere. Subsequently, the cells were exposed to 365 nm UV light for 4 min using a 365 nm UV lamp (high performance UV transilluminator, UVP, 25 W) or kept in the dark. Expression level of the Cre activation reporter was measured after 24 h incubation.

Spatial control of Cre Activation. In order to demonstrate spatial control of light-activated Cre-**PCK** via locally controlled light irradiation in living cells, HEK293T cells expressing both the caged Cre recombinase and the pC-SL reporter in a 35 mm imaging μ-dish (observation area 21 mm, ibidi) were exposed to UV irradiation in a "smiley face" pattern, followed by microcopy and ChemiDoc (Bio-Rad) imaging. Bright EGFP fluorescence was only observed for cells containing activated Cre recombinase in the patterned region. Non irradiated cells were dark. All cells showed DsRed expression, as a control. In response to different UV exposure times, different EGFP fluorescence intensity was observed in right eye (5 min's irradition) and the left eye and mouth (3.5 min's exposure), demonstrating tuning of Cre decaging and activation.

References

(1) Edwards, W. F.; Young, D. D.; Deiters, A. *ACS Chem Biol* **2009**, *4*, 441.

(2) Arbely, E.; Torres-Kolbus, J.; Deiters, A.; Chin, J. W. *J Am Chem Soc* **2012**, *134*, 11912.
